# Lymphatic vessels assessment in feline mammary tumours

**DOI:** 10.1186/1471-2407-7-7

**Published:** 2007-01-12

**Authors:** Giuseppe Sarli, Francesco Sassi, Barbara Brunetti, Antonio Rizzo, Laura Diracca, Cinzia Benazzi

**Affiliations:** 1 Department of Veterinary Public Health and Animal Pathology, Via Tolara di Sopra, 50 – 40064 Ozzano Emilia – Bologna – Italy

## Abstract

**Background:**

The lymphatic vessels play a crucial role in a variety of human cancers since tumour cell lymphatic invasion significantly influences prognosis. It is not known if pre-existing lymphatics are enough for tumour dissemination or *de novo *development is necessary. VEGFR-3 is an angiogenetic mediator for both lymphatic and blood vessels during embryonic development, and only for lymphatics after birth. VEGF is a mediator of both vasculogenesis and angiogenesis, regulates the growth of lymphatics in various experimental models, and is produced in many solid tumours. CD44 mediates hyaluronic acid (HA)-dependent cell adhesion: besides promoting invasion, this interaction also supports neoangiogenesis that indirectly stimulates tumour cell proliferation. The expression of VEGF-C (Vascular Endothelial Growth Factor – C), its receptor VEGFR-3 and CD44, were studied on feline mammary samples to assess the importance of lymphangiogenesis and lymphangiotrophism in neoplasia.

**Methods:**

Samples were taken from six normal mammary glands (NMG), ten benign (BT) and 32 malignant (MT) tumours. Immunohistochemical laminin/VEGFR-3 double stain, VEGF-C and CD44 stains were applied to 4 μm-thick sections, and their expression evaluated in intratumoral/extratumoral and intramammary/extramammary fields.

**Results:**

All groups revealed a higher number of lymphatics in the extratumoral/extramammary areas. VEGF-C expression in the epithelium paralleled the number of positive vessels in the NMG, BT and MT, whereas VEGF-C higher expression was noted in the intratumoral fields only in infiltrating MT. CD44 score was lower in extratumoral than intratumoral fields in tumours and showed a significant increase in extramammary/extratumoral fields from NMG to MT. Pearson test showed a significant and inversely proportional correlation between CD44 expression and the number of lymphatic vessels with VEGFR-3 in malignant infiltrating tumours.

**Conclusion:**

The number of both VEGFR-3 positive and negative lymphatics in the extratumoral and extramammary stroma was significantly higher than intratumoral and intramammary fields respectively in the NMG, BT and MT. This suggests a scant biological importance of intratumoral lymphatics while their higher number is due to the concentration of existing vessels following compression of the extratumoral stroma in spite of a non demonstrable increase from NMG to MT. The tumour model employed provided no evidence of lymphangiogenesis, and metastasis in the regional lymph node develops following the spread through the pre-existing lymphatic network.

## Background

The lymphatic vessels play a crucial role in a variety of human cancers since tumour cell lymphatic invasion and subsequent development of lymph node metastases significantly influences prognosis. Therefore the lymphatic pathway is an integral part of tumour staging [1].

It is well established that for many carcinomas transport of tumour cells via the lymphatics is the most common pattern of initial dissemination. However, it is not known if pre-existing lymphatics are enough for tumour dissemination or *de novo *development is necessary [2].

Until recent years, the lack of specific markers has raised problems in documenting exactly how the lymphatic system develops. The markers recently identified are the homeobox gene Prox-1, involved in the early phases of lymphatic vessel development [3]; Podoplanin, a 43 kDa mucoprotein found on the membrane of glomerular podocytes [4]; LYVE-1 (Lymphatic vessel endothelial hyaluronan receptor-1), a lymphatic endothelial receptor for hyaluronan, the extracellular matrix/lymphatic fluid glycosaminoglycan [5].

These markers seemed to have solved the problem of detecting the lymphatic vessels and, above all, their histological distinction from blood vessels. However, these molecules are not always detectable in animal tissues, and need to be used with specific blood vessel markers to discriminate between the two vessel types. Many studies highlighted the limits of these markers: i.e. LYVE-1 is present not only in lymphatic vessels, but also in the hepatic blood sinusoids [6], with a variable behaviour according to the tumour type [5]. Podoplanin is not highly specific, since it is also expressed by glomerular podocytes. Prox-1 has an important role in the embryonic development of lymphatic vessels [3], but is not clearly expressed in lymphatics after birth or in adults [7].

The role of VEGFR-3 (Vascular Endothelial Growth Factor Receptor-3) as an angiogenetic mediator was demonstrated for both lymphatic and blood vessels during embryonic development, and only for lymphatics (lymphangiogenesis) after birth [8]. VEGFR-3 is the receptor for VEGF-C, and belongs to the vascular endothelial growth factor (VEGF) protein family. VEGF, discovered in 1989, is a major mediator of both vasculogenesis and angiogenesis. VEGF-C regulates the growth of lymphatics in various experimental models [9], and is produced in many solid tumours [10]. Its level of expression has been suggested to correlate with angiogenesis and metastasis via the lymphatic system [11].

CD44 is a broadly distributed transmembrane glycoprotein playing a critical role in a variety of cellular behaviours, including adhesion, migration, invasion, and survival [12]. CD44 mediates hyaluronic acid (HA)-dependent cell adhesion [13]: besides promoting invasion, this interaction also supports neoangiogenesis that indirectly stimulates tumour cell proliferation [14].

Our investigation employed an animal model known to use the lymphatic pathway to metastasize, i.e. feline mammary carcinoma, to assess the importance of lymphangiogenesis as a step in this tumour model for the spread of neoplastic cells through the lymphatics instead of blood vessels.

## Methods

### Cases

The samples used originated from 48 cases: six normal mammary glands, ten benign tumours (tubulo-papillary adenoma) and 32 malignant (six *in situ *carcinomas, 12 tubulo-papillary carcinomas and 14 solid carcinomas) of the mammary gland of female cats. Ethical approval for the study was not required because cases were selected among those routinely collected at the laboratory of histopathology for diagnostic purposes after mastectomy.

Each specimen had been formalin-fixed and paraffin-wax embedded and histological diagnoses were performed on Haematoxylin-Eosin stained sections according to the WHO criteria by Misdorp et al. [15]. Malignancies were then graded according to the histological staging method developed by Gilbertson et al. [16] in the dog and adapted to the cat by Mandelli et al. [17] as follows: stage 0 (St0) or malignant non-infiltrating tumour, when the neoplastic proliferation was limited by the basement membrane (all these cases belong to the *in situ *carcinomas of the WHO [15] classification); stage I (StI) or infiltrating malignant tumour with stromal invasion (i.e. the tumour proliferated beyond the basement membrane and invaded the surrounding stroma); stage II (StII) or infiltrating malignant tumour with lymphatic or blood emboli and/or regional lymph node metastases. On the basis of this gradation system, 6 malignancies resulted stage 0 (*in situ*), 14 stage I and 12 stage II.

### Immunohistochemistry

Four μm-thick sections from all the samples were immunohistochemically stained with a laminin/VEGFR-3 double stain, VEGF-C and CD44 stains.

### Laminin/VEGFR-3 double stain reaction

Sections of formalin-fixed and paraffin-embedded samples were dewaxed and rehydrated. Antigen was unmasked with 0.05% Pronase E (1.07433, Merck, Darmstadt, Germany) in Tris buffer pH 7.4 for 10 min at room temperature followed by citrate buffer (2.1 g citric acid monohydrate/liter distilled water) pH 6.0 in a microwave oven at 750 W (two cycles of 5 min each); then the sections were allowed to cool at room temperature for 20 min. The first immunohistochemical step was the block of peroxidase reagent with DAKO EnVision™ Doublestain System (Dako, Amsterdam, The Netherlands) for 5 min followed by primary polyclonal rabbit anti-mouse laminin antibody (Sigma, St. Louis, Missouri, USA), diluted 1:200 in PBS containing 1% bovine serum albumin applied overnight at 4°C. After a wash with TRIS buffer the peroxidase labelled components (diaminobenzidine as chromogen) of the DAKO EnVision™ Doublestain System were used to reveal this part of the double immunohistochemical stain. After a step with double staining block solution of the DAKO EnVision™ Doublestain System, the primary polyclonal rabbit anti-mouse FLT-4/VEGFR-3 antibody (Alpha Diagnostic International, San Antonio, Texas, USA) diluted 1:350 in PBS containing 1% of bovine serum albumin was applied for 60 min at room temperature. After a wash with TRIS buffer the alkaline phosphatase labelled components (fast red as chromogen) of the DAKO EnVision™ Doublestain System were used to reveal this second part of the double immunohistochemical stain. Apart from primary antibodies, all other steps were conducted following the kit manufacturer's instructions. Sections were counterstained with Mayer's hematoxylin and mounted with DAKO Glycergel Mounting Medium (Dako, Amsterdam, The Netherlands). Negative controls were achieved by incubating the slides with a non-specific antibody of the same isotype. As positive controls to assess the cross-reactivity with feline tissues and the specificity of the immunohistochemical stain of the anti-VEGFR-3 antibody a section of feline intestine was used with the same protocol, employing one of the two immunohistochemical DAKO EnVision™ Doublestain System steps. The specificity and cross-reactivity of the anti-laminin stain were evaluated as expected in basement membranes surrounding glandular structures of NMG, BT and MT, and around blood containing vessels.

### VEGF-C and CD44 stains

Four μm-thick sections were dewaxed in toluene and rehydrated. Endogenous peroxidase was blocked by immersion in 0.3% hydrogen peroxide for 20 min. Sections were then rinsed in Tris Buffer and antigen retrieval was obtained only for anti-CD44 stain with citrate buffer (2.1 g citric acid monohydrate/liter distilled water), pH 6.0, and heating for two 5-min periods in a microwave oven at 750 W, followed by cooling at room temperature for 20 min. Sections were then preincubated with serum free protein block (Dako, Amsterdam, The Netherlands) for 10 min at room temperature. The primary antibodies anti-VEGF-C, clone Z-CVC7, diluted 1:12.5 (Zymed laboratories inc., San Francisco, California, USA) and anti-CD44var (v5), clone VFF-8, diluted 1:12.5 (Bender MedSystems, Vienna, Austria) were applied overnight at 4°C and were followed by a commercial streptoavidin-biotin-peroxidase technique (LSAB Kit, Dako, Amsterdam, The Netherlands). Diaminobenzidine (0.05% for 10 min at room temperature) was used as chromogen. Slides were counterstained with Papanicolaou's hematoxylin. Negative controls were obtained substituting the primary antibody with an unrelated monoclonal antibody of the same isotype. As positive controls to assess the cross-reactivity with feline tissues and the specificity of the immunohistochemical stain of the anti-VEGF-C and anti-CD44 antibodies, a section of granulation tissue from a feline healing skin lesion and one from a feline anaplastic mammary carcinoma respectively were used following the same protocols.

### Scoring methods

Immunohistochemical expression was evaluated in intratumoral (fields randomly chosen between lobules inside the tumour) and extratumoral areas (between the external border of the neoplasm and the subcutis or the muscle/stromal plan of the abdomen or thorax). Intramammary and extramammary fields were selected in the normal mammary gland considering any area containing normal lobuli and/or interlobular connective tissue in the first case, and the subcutis or the muscle/stroma plan of the abdomen or thorax in the second.

Lymphatic vessels expressing and not expressing VEGFR-3 were counted in ten intratumoral/intramammary fields and 20 extratumoral/extramammary fields. Each field had an area of 0.789 square mm and the microscope objective used was 20x. Counts are expressed as the number of lymphatic vessels in ten square millimitres.

VEGF-C and CD44 immunohistochemical expressions were analyzed with a 40x objective (corresponding to 0.196 mm^2^) on five intratumoral (intramammary) and ten extratumoral (extramammary) fields, selecting the areas with major expression (Hot Spots) at low magnification. In each immunostained area, VEGF-C and CD44 were expressed as score index of the epithelial, adenomatous and carcinomatous components as follows: absent = 0, i.e. no positive cells per field; low = 10, up to ten positive cells per field; intermediate = 100, fewer than 50% positive cells per field; high = 1000, more than 50% positive cells per field. Only for VEGF-C were the number of positive vessels in the same field also counted.

### Statistical analysis

The variables (number of lymphatics expressing and not expressing VEGFR-3, VEGF-C and CD44 scores, and number of VEGF-C expressing vessels) were tested for normality with Shapiro Wilk's W Test and the data did not show a normal distribution. Comparison between cases grouped as for diagnosis (normal mammary gland, benign and malignant tumours), site of evaluation (intratumoral/intramammary, extratumoral/extramammary) and stage of invasion (non infiltrating, locally invasive (StI) and with intravascular emboli (StII)) were evaluated with the Spearman rank test. Pearson's correlation test was employed to examine the variables relationship. Analyses were performed by CSS software (Statsoft, Tulsa, OK, USA) statistics, and a conventional 5% level was used to define statistical significance.

## Results

### Immunohistochemical anti-laminin/anti-VEGFR3 double stain

**Figure 1:**
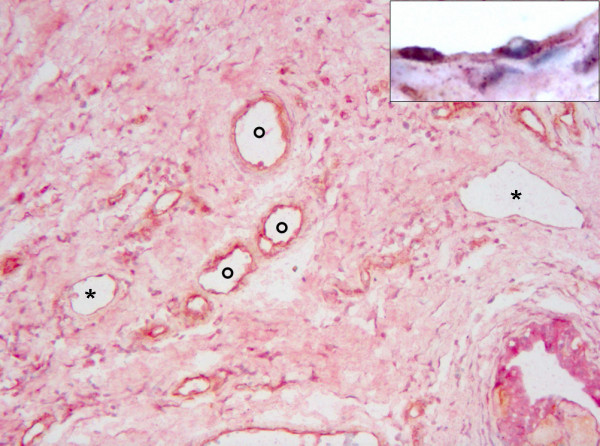
Cat. Mammary gland. VEGFR-3 (red) and laminin (brown) double immunohistochemical stain in an extratumoral area of a stage 0 carcinoma. Lymphatic vessels are laminin negative (asterisk) whereas blood vessels are laminin positive (circle) 20X. Inset: higher magnification of VEGFR-3 positive endothelial cells in a lymphatic vessel. 63X.

Laminin in the basement membrane stained brown (depending on the chromogen used), and VEGFR-3 intense red (figure 1). Laminin stain was also visible in the sub-endothelial space of blood containing vessels around alveoli and ducts in NMG and around tubules and papillae in BT and most MT. VEGFR-3 in controls was revealed as a granular cytoplasmic stain in some endothelial cells of the lacteals and in some blood containing and non-blood containing vessels of the intestinal wall. Lymphatic vessels were recognized when laminin stain was unappreciable (absence of the basement membrane), associated or not with VEGFR-3 in the cytoplasm of the lining endothelium. Blood capillaries were recognized when continuous positive laminin stain was present, associated or not with positive VEGFR-3 red staining in the cytoplasm of the lining endothelium. This study reports only the results of lymphatics quantification. VEGFR-3 expression stained positive both in the blood and lymphatic endothelium, in the epithelium of normal mammary gland and in benign and malignant tumours.

**Figure 2:**
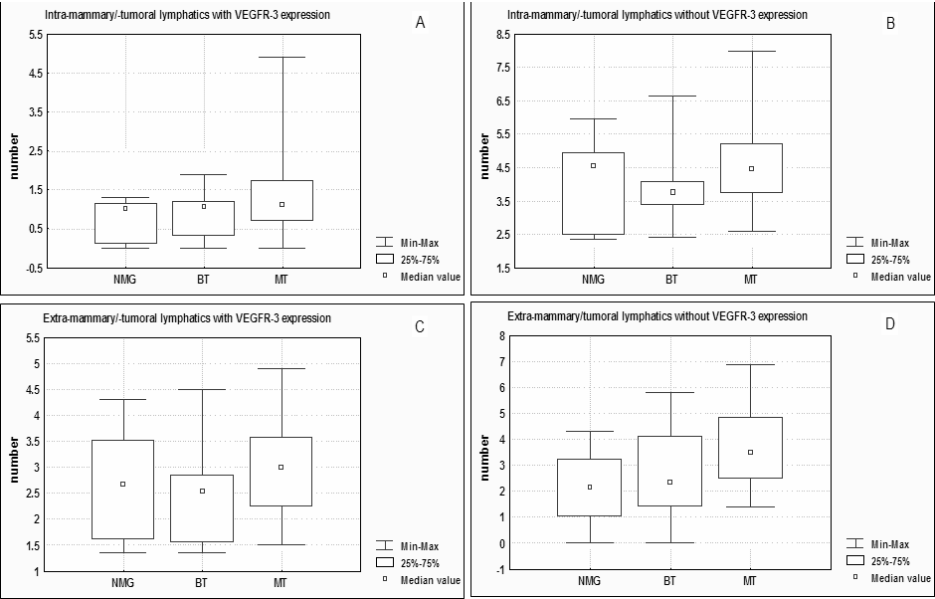
Number of lymphatics with and without VEGFR-3 expression in intramammary/intratumoral (A, B) and extramammary/extratumoral (C, D) fields in normal mammary gland (NMG), benign tumours (BT) and malignant tumours (MT).

**Figure 3:**
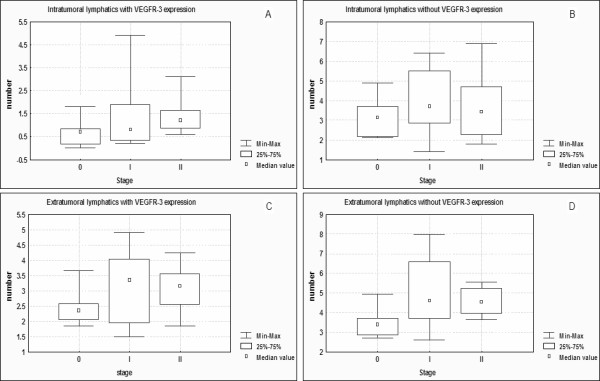
Number of lymphatics with and without VEGFR-3 expression in intratumoral (A, B) and extratumoral (C, D) fields in stage 0, stage I and stage II malignant tumours.

Descriptive statistical data of the number of lymphatics with or without VEGFR-3 expression in the groups compared are reported in table 1. The number of total lymphatic vessels (i.e. those expressing and non expressing VEGFR-3) was significantly higher in the extratumoral/extramammary than intratumoral/intramammary areas (Spearman test: P < 0.01 for NMG and MT; P < 0.05 for BT). Both numbers of lymphatics with or without VEGFR-3 expression in NMG and carcinomas had higher significant values in extramammary/extratumoral fields compared to intramammary/intratumoral stroma (Spearman test; NMG: P < 0.05 for lymph vessels without and P < 0.01 for those with VEGFR-3 expression; MT: P < 0.05 for lymph vessels without, and P < 0.01 for those with VEGFR-3 expression), whereas in adenoma the same result was obtained for lymphatic vessels with VEGFR-3 (Spearman test: P < 0.01). No significant difference was disclosed in the number of lymphatics without VEGFR-3 expression (figure 2). A significantly higher number of lymphatics without VEGFR-3 expression from NMGs to MTs (Spearman test: P < 0.05) in the extratumoral (extramammary) fields, but no significant difference in the intratumoral (intramammary) fields was found in the three diagnostic groups (normal mammary gland, benign and malignant tumours). Grouping malignant tumours according to stage, no significant difference was found in the extratumoral stroma, and a significant increase in the number of lymphatics expressing VEGFR-3 in the intratumoral stroma from stage 0 and I vs stage II (median values: stage 0 = 0.7; stage I = 0.8; stage II = 3.3; Spearman test: P < 0.05) (figure 3).

### Immunohistochemical anti-VEGF-C stain

**Figure 4:**
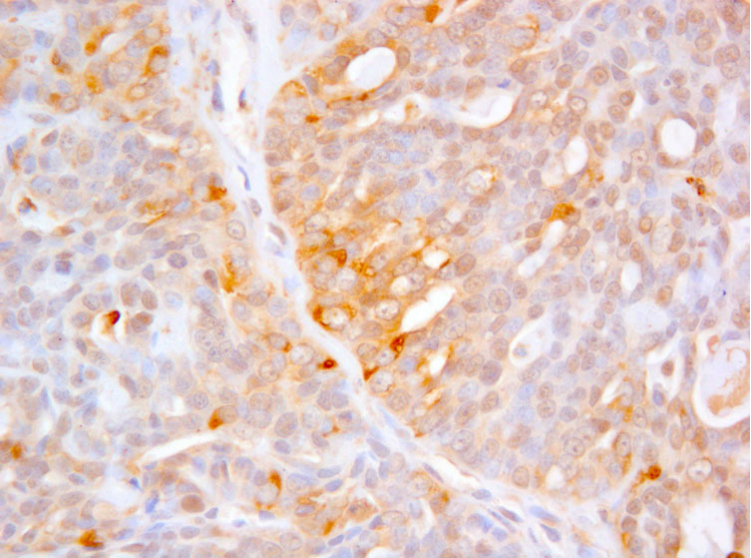
Cat. Mammary gland. Strong cytoplasmic and light nuclear VEGF-C positivity in cells of solid carcinoma. 40X.

Vascular endothelium and stromal fibroblasts in positive controls and also the epithelium of both NMG, BT and MT, in mammary samples showed a brown stain, strong in the cytoplasm and light in the nucleus (figure 4). Stained cells were located in "hot spots" in both benign and malignant neoplasms.

**Figure 5:**
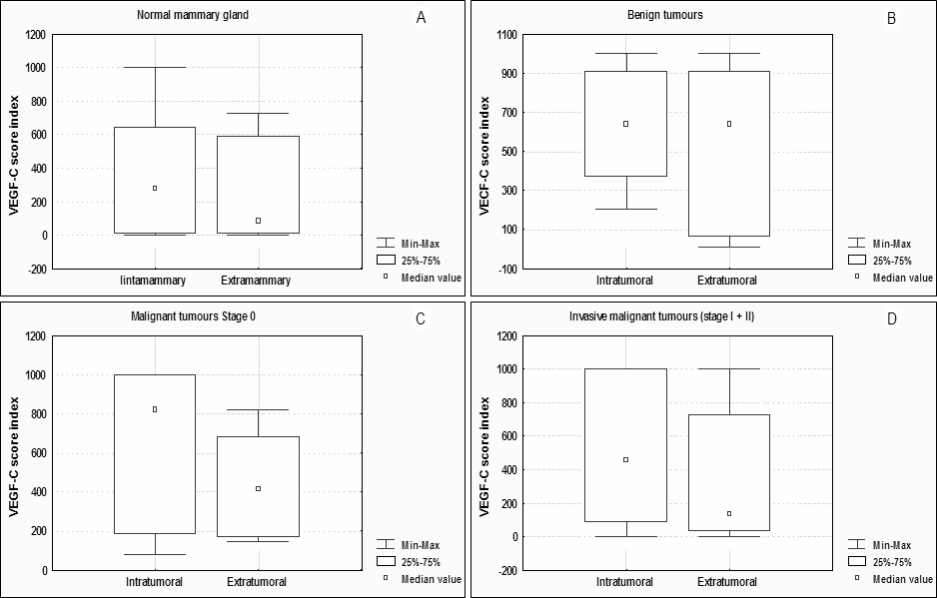
VEGF-C score index in normal mammary gland (A), benign tumours (B), non-invasive (C) and invasive malignant (D) tumours.

Descriptive statistical data of the VEGF-C score index in the comparison between groups are reported in table 2. A significant association was found between VEGF-C expression in the epithelium and the number of positive vessels (Pearson correlation, R = 0.67, P < 0.01) both in the normal mammary gland, and benign and malignant tumours. In the malignant tumours (Spearman: P = 0.024), but not in the benign lesions (Spearman: P = 0.46) or in the normal mammary gland (Spearman: P = 0.62), the VEGF-C score index had higher and significant values in the intratumoral (intramammary) than in extratumoral (extramammary) fields (figure 5). Comparing the expression of VEGF-C of the epithelial component between malignant infiltrating (St I+II) and non-infiltrating tumours (St0), a higher significant difference was noted in the intratumoral than extratumoral fields in infiltrating tumours (Spearman: P = 0.036), but not in non-infiltrating neoplasms (Spearman: P = 0.31) (figure 5).

No relationship was found between the VEGF-C score in epithelial cells and the diagnostic group (normal mammary gland, benign and malignant tumours) or histologic stage (0, I, II).

### Immunohistochemical anti-CD44 stain

**Figure 6:**
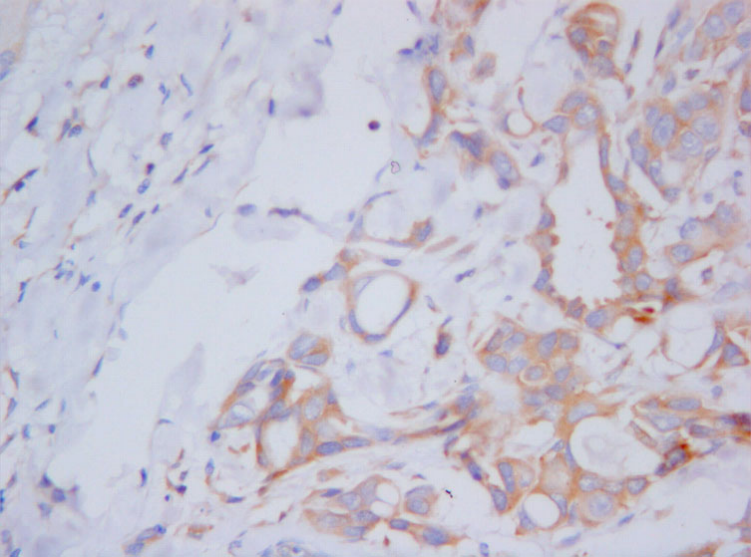
Cat. Mammary gland. CD44 cytoplasmic positivity of carcinoma infiltrating cells close to a lymph vessel. 40X.

CD44 was immunohistochemically disclosed as a brown cytoplasmic stain in the positive control (feline anaplastic mammary carcinoma cells) and in the epithelium of NMG as well as BT and MT (figure 6). The immunostaining was concentrated in "hot spots" where the quantitative analysis was focused both for normal mammary gland and benign and malignant growths.

Descriptive statistical data of the CD44 score in the groups compared are reported in table 3.

**Figure 7:**
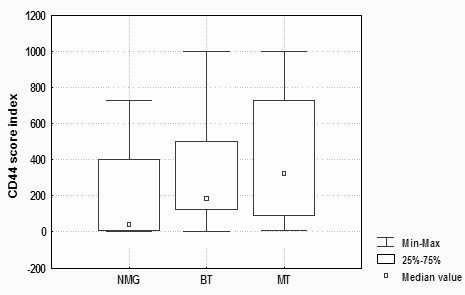
CD44 score index in extramammary/extratumoral fields of NMG, BT and MT.

CD44 score was significantly lower in extratumoral than intratumoral fields in both benign and malignant tumours (Spearman: P < 0.01). The normal mammary gland showed values close to significance (Spearman: P = 0.056). The comparison of intramammary/intratumoral and extramammary/extratumoral fields showed a significant increase in CD44 score in extramammary/extratumoral fields from NMG to MT (Spearman: P < 0.05) (figure 7) but not in intramammary/intratumoral stroma. The CD44 index was not correlated with stage progression in MT.

### Correlations of lymphatic assessment with VEGF-C and CD44 indices

The expression of VEGF-C and CD44 in the epithelial neoplastic cells was compared with the number of lymphatic vessels with or without VEGFR-3 expression. Pearson test showed a significant and inversely proportional correlation between CD44 expression and the number of lymphatic vessels with receptor in malignant infiltrating tumours (StI + II) (R = -0.36; P = 0.18), but not in non-infiltrating malignant tumours, benign tumours and normal mammary gland.

## Discussion

On the basis of the results obtained, the distribution of the lymphatic vessels produced quantitative data in the intratumoral and extratumoral stroma of benign and malignant tumours similar to those of the intra- and extramammary areas of the normal mammary gland. In all groups (normal mammary gland, benign tumours and malignant tumours) the number of both VEGFR-3 positive and negative lymphatics in the extratumoral (extramammary) stroma was significantly higher than that in the intratumoral (intramammary) stroma.

These data emphasize in the cat, as in the human, there are only a few lymphatic vessels in the intratumoral stroma. This suggests a scant biological importance of the intratumoral lymphatics that do not seem to be invaded by neoplastic cells because of the high interstitial pressure inside the tumour. The lymphatics are instead better represented in the extratumoral tissue where, as in the woman, the mechanical stress is less intense, and presumably due to the compression of the stroma in these areas of concentration of the pre-existing vessels.

Like the extratumoral and intratumoral environment of benign and malignant tumours, the lymphatics expressing VEGFR-3 in the extramammary tissue of normal mammary gland are about threefold those in the intramammary stroma. The results of the normal mammary gland and intra/extratumoral tissue obtained in this study suggest that a new production of lymphatic vessels or lymphangiogenesis is extremely limited. No significant increase in the number of lymphatic vessels expressing VEGFR-3 was detected in extratumoral tissue. The lack of an increase in lymphatic vessels demonstrated at the tumour periphery fails to explain why early metastases, especially in the case of mammary neoplasms, are localized in the regional or collector lymph node, proving that the neoplastic cells leaving the primary tumour spread via the lymphatic pathway. In this respect, some chemical mediators or ligand-receptor interactions (i.e. VEGF ligands and VEGF receptors) might direct the neoplastic cells to the lymphatic vessels.

In relation to tumour stage, VEGFR-3 expression in lymphatics was correlated with the histological stage of carcinomas, with a specific increase in VEGFR-3 expression in the intratumoral stroma from St0 to StI and to StII. VEGFR-3 expression is thought to be strictly related to the formation of tumour metastasis and, consequently, to the patient's prognosis. Human patients with the highest relative number of VEGFR-3 positive lymphatics and the lowest relative number of blood vessels positive for the same receptor had the highest probability of lymph node metastases and therefore a negative prognosis [1]. The presence of VEGFR-3 negative lymphatics is likely to indicate a greater difficulty of neoplastic emboli entering negative rather than positive vessels for this receptor. Even if results on haematic vessels are not reported in this paper, many blood vessels were VEGFR-3 positive in intratumoral fields, demonstrating their recent formation. In fact, the expression of this receptor by the endothelium of tumoral blood vessels is well known, suggesting that VEGFR-3 is involved in the angiogenesis of tumours and in tumorigenesis [18,19,11]. The scant lymphangiogenesis inside the tumours may indicate that neoplastic cells developing in a confined space create a mechanical stress compressing the lymphatics or inhibiting their new formation inside the tumour [20]. By contrast, blood vessel angiogenesis would not be inhibited by major wall resistance, but more likely by a local difference in the balance of cytokines stimulating and inhibiting lymphangiogenesis.

Immunohistochemical VEGF-C positivity was observed both in the cytoplasm and nucleus for the first time by clone Z-CVC7 in the cat. Besides having a positive trophic influence on vessel endothelium like other components of the same family [21], anti-VEGF-C immunohistochemistry also stained epithelial cells. The data obtained comparing the expression of the epithelial component with the number of positive vessels showed that the two are significantly associated. The factors inducing genic expression of VEGF-C in the endothelial wall, such as pro-inflammatory cytokines (IL-1β and Tumour Necrosis Factor) [22,23], are also likely to influence epithelial cells. Apart from the proportional relationship between the increase in VEGF-C positivity of the epithelium and the number of positive vessels, no relation was found between the number of positive vessels and the diagnostic group (comparison between normal mammary gland, benign and malignant tumours), evaluation field (intratumoral/intramammary or extratumoral/extramammary) and histological stage.

Based on the expression of VEGF-C of the epithelial component, both normal gland and benign tumours produced similar results, for example regarding the comparison between intratumoral (intramammary) and extratumoral (extramammary) fields. The expression was significantly higher in intratumoral than extratumoral areas only in malignant tumours. The conditions stimulating angiogenesis are likely to act in the intratumoral areas due to mechanical stress [20] more than at the periphery of the neoplasm where the vasculature is accentuated. The same behaviour was observed in malignant tumours grouped according to stage, for infiltrating (StI and StII) but not for non-infiltrating carcinomas. The enhanced intratumoral expression in infiltrating lesions reflects their more pronounced angiogenetic need.

VEGF-C expression is not limited to the vascular endothelium, but also regards the epithelium of normal and neoplastic gland. The inductive stimuli on the endothelium may also induce ligand expression on the epithelium. This expression is evident above all in infiltrating malignancies, and especially in intratumoral hypoxic areas.

The results of VEGF-C were reproduced with CD44, with enhancement of statistically significant differences, especially in benign and malignant tumours. Only stage I and II infiltrating malignancies showed a different expression in the intratumoral and extratumoral areas, being significantly more pronounced in the intratumoral than extratumoral stroma. Different roles have been attributed to CD44, such as cell adhesion, lymphocytic homing, growth signals transmission and acquisition of invasive malignant phenotype [24]. It is well-known that the degradation of hyaluronic acid (a structural protein of the extracellular matrix) plays an important role in tumour invasion. CD44-mediated degradation of hyaluronic acid around vessels allows tumour cells expressing the adhesion molecule to enter the flow quickly and develop metastases [25]. The higher values of CD44 expression in the intratumoral hypoxic areas, especially in infiltrating malignant tumours, are also likely to reflect such biological functions in the neoplastic model.

The CD44-dependent endothelium-lymphocytes interaction takes place only if lymphocytes are activated and the endothelium stimulated by IL-2 [26]. This points out that endothelial activation may be fundamental for entering vessels. Following an activation signal, VEGFR-3 is expressed by the vessel endothelium (VEGFR-3 is not always expressed in all vessels). This may indicate neoplastic CD44 expressing cells are the means for entering vessels thanks to an interaction with activated endothelium. The inverse proportion between CD44 expression in tumour cells and the number of lymphatics expressing VEGFR-3 may be due to the role of CD44 in homophilic cell aggregation. CD44-dependent cell aggregation has been widely described [24]. Spontaneous homophilic aggregation of many cell lines presenting CD44 depends on the presence of this ligand that probably promotes the adhesion of these cells [24]. In the pathogenesis of tumoral progression, cell aggregation may protect metastatic cells from the immunosurveillance mechanisms facilitating their entrapment in target organs [24]. The inverse relationship deriving from the results of the current investigation is present in two different sites: intratumoral with high CD44 expression in the tumour cells together with a low number of VEGFR-3 expressing lymphatics, and extratumoral showing the opposite. Extratumoral areas with scant CD44 (low efficient homotypic adhesion), and the higher number of VEGRF-3 expressing lymphatics (maximum preferential interaction of the neoplastic cell and the endothelium) would allow the neoplastic cells to enter the lymphatic pathway.

## Conclusion

The tumour model employed in the present study provided no evidence of lymphangiogenesis, i.e. an increase in the number of newly formed lymphatic vessels that could have been proven by the increased expression of VEGFR-3. In any case, it is likely that neoplastic cells reach the regional lymph nodes following spread through the pre-existing lymphatic network.

## Competing interests

The author(s) declare that they have no competing interests.

## Authors' contributions

CB and GS conceived the study, participated in its design and coordination and drafted the manuscript. BB supervised the immunohistochemical stains and helped co-authors in performing image analysis: AR (anti-VEGFR-3 and anti-laminin double stain), LD (anti-VEGF-C stain) and FS (anti-CD44 stain). GS performed the statistical analysis. All authors read and approved the final manuscript.

## Pre-publication history

The pre-publication history for this paper can be accessed here:



## Figures and Tables

**Table 1:** VEGFR-3 expression in lymphatics of normal mammary gland, benign and malignant tumours.

Groups	N° of lymphatics		Spearman test
		
	without VEGFR-3 (mean ± st. err; median)	with VEGFR-3 (mean ± st. err; median)	
Normal mammary gland			^a^vs^b ^**
intramammary	2.43 ± 0.54; 2.16^a^	0.79 ± 0.18; 1.03^c^	^c^vs^d ^**
extramammary	4.03 ± 0.53; 4.55^b^	2.75 ± 0.41; 2.66^d^	^e^vs^f ^NS
	
Benign tumours			^g^vs^h ^**
intratumoral	2.80 ± 0.66; 2.33^e^	0.93 ± 0.21; 1.05^g^	^i^vs^l ^**
extratumoral	4.20 ± 0.49; 3.76^f^	2.64 ± 0.36; 2.53^h^	^m^vs^n ^**
	
Malignant tumours			^a^vs^e^vs^i ^NS
intratumoral	3.76 ± 0.27; 3.50^i^	1.30 ± 0.18; 1.10^m^	^b^vs^f^vs^l ^*
extratumoral	4.55 ± 0.22; 4.44^l^	3.03 ± 0.15; 3.01^n^	^c^vs^g^vs^m ^NS ^d^vs^h^vs^n ^NS

Malignant tumours intratumoral			
Stage 0	3.25 ± 0.44; 3.15^o^	0.73 ± 0.25; 0.70^r^	
Stage I	4.07 ± 0.47; 3.70^p^	1.43 ± 0.42; 0.80^s^	^o^vs^p^vs^q ^NS
Stage II	5.06 ± 0.51; 4.58^q^	3.20 ± 0.34; 3.35^t^	^r^vs^s^vs^t ^*
	
Malignant tumours extratumoral			^u^vs^v^vs^w ^NS
Stage 0	3.52 ± 0.32; 3.37^u^	2.52 ± 0.25; 2.35^x^	^x^vs^y^vs^z ^NS
Stage I	5.06 ± 0.51; 4.58^v^	3.20 ± 0.34; 3.35^y^	
Stage II	4.59 ± 0.17; 4.52^w^	3.12 ± 0.18; 3.15^z^	

Descriptive statistical data of the number of lymphatics with or without VEGFR-3 expression in the compared groups. Spearman test: * = P < 0.05; ** = P < 0.01; NS = non-significant.

**Table 2:** VEGF-C expression in normal mammary gland, benign tumours, non-invasive and invasive malignant tumours.

Groups	VEGF-C score index (mean ± st. err; median)	Spearman Test
Normal mammary gland		
intramammary	381.1 ± 437.4; 280.0^a^	^a^vs^b ^NS
extramammary	292.3 ± 336.6; 82.0^b^	
Benign tumours		
intratumoral	671.3 ± 309.5; 640.0^c^	^c^vs^d ^NS
extratumoral	530.1 ± 446.21; 630.0^d^	
Non-invasive malignant tumours		
intratumoral	667.0 ± 165.8; 820.0^e^	^e^vs^f ^NS
extratumoral	451.0 ± 127.1; 415.0^f^	
Invasive malignant tumours		
intratumoral	501.16 ± 83.5; 460.0^g^	^g^vs^h ^*
extratumoral	284.2 ± 70.6; 82.0^h^	

Descriptive statistical data of VEGF-C score index in the compared groups. Spearman test: * = P < 0.05; NS = non-significant.

**Table 3:** CD44 expression in normal mammary gland, benign and malignant tumours.

Groups	CD44 score index (mean ± st. err; median)	Spearman Test
Normal mammary gland		
intramammary	720.8 ± 148.9; 1000^a^	^a^vs^b ^NS
extramammary	227.1 ± 130.1; 37^b^	
Benign tumours		
intratumoral	889.1 ± 110.9; 1000^c^	^c^vs^d ^**
extratumoral	379.9 ± 122.5; 181.1^d^	
Malignant tumours		
intratumoral	762.8 ± 65.8; 1000^e^	^e^vs^f ^**
extratumoral	438.1 ± 62.6; 320.5^f^	
		^a^vs^c^vs^e ^NS ^b^vs^d^vs^f ^*

Descriptive statistical data of CD44 score index in the compared groups. Spearman test: * = P < 0.05; ** = P < 0.01; NS = non-significant.
